# Inflammation, Oxidative Stress, and Antioxidant Micronutrients as Mediators of the Relationship Between Sleep, Insulin Sensitivity, and Glycosylated Hemoglobin

**DOI:** 10.3389/fpubh.2022.888331

**Published:** 2022-06-09

**Authors:** Thirumagal Kanagasabai, Michael C. Riddell, Chris I. Ardern

**Affiliations:** School of Kinesiology and Health Science, York University, Toronto, ON, Canada

**Keywords:** mediation effect, sleep duration, sleep quality, fasting insulin, inflammation, oxidative stress, antioxidant micronutrients

## Abstract

**Background:**

Sleep deprivation and poor sleep quality contribute to increases in oxidative stress, antioxidant imbalance, and a pro-inflammatory state which may predispose to a higher risk of diabetes. Our objective was to estimate the contributions of C-reactive protein (CRP), gamma glutamyl transferase (GGT), and micronutrient antioxidants (bilirubin, carotenoids, uric acid, vitamins A, C–E?) to the relationships between sleep-fasting insulin concentration and -glycosylated hemoglobin (HbA1c).

**Methods:**

Data from the 2005/06 US National Health and Nutritional Examination Survey were used (*N* = 1,946; 20 y+). Sleep quality and quantity was assessed by the Sleep Disorders Questionnaire, and fasting blood was collected to quantify CRP, GGT, antioxidant micronutrients, insulin concentration, and HbA1c. The bootstrap method was used to estimate the amount of mediation or contribution of these mediators to the sleep–insulin concentration and -HbA1c relationships, which were quantified as large (≥0.25) or moderate (≥0.09).

**Results:**

The sleep *duration*–fasting insulin relationship was mediated by GGT, carotenoids, uric acid, and vitamins C and D, whereas CRP and bilirubin were non-significant mediators of a moderate effect size. Similarly, the sleep *quality*–fasting insulin relationship was mediated by CRP, bilirubin and vitamin C, whereas GGT, carotenoids, uric acid, and vitamin D were non-significant large-to-moderate mediators. To a lesser degree, these micronutrients mediated for the relationship between sleep-HbA1c levels.

**Conclusion:**

Several factors related to inflammation, oxidative stress, and antioxidant status were found to lie on the pathway of the sleep–insulin and –glycemic control relationships. Sleep hygiene, reduced systemic inflammation/oxidative stress, and optimal antioxidants intake are potentially beneficial targets for managing diabetes risk.

## Introduction

Sleep is vital for optimal health, but most adults do not obtain the recommended 7–9 hours of sleep per night on a regular basis (1). Sleep duration also shares a complex relationship with sleep quality, which modifies its associations with health (2). Poor sleep quantity and quality have been associated with several physiological and pathological conditions, including cardiovascular, neurological, endocrine, and immunological disorders (3). Sleep deprivation, for example, is known to affect cardiovascular disorders by impairing autonomic function, altering glucose and lipid metabolism, and increasing the risks of atherosclerosis and ischemia (3). Further, subchronic sleep restriction may increase peripheral insulin resistance without affecting hepatic insulin sensitivity (4), while sleep extension may improve fasting insulin sensitivity (5). Poor sleep is also associated with poor diets and behaviors that compromise overall health (6, 7).

A growing body of work supports an association between inflammation, oxidative stress, and antioxidant micronutrients with sleep (8–13), including two of our own cross-sectional studies (8, 9). In this most recent work, we quantified the contributions of inflammation [i.e., C-reactive Protein (CRP)], oxidative stress [i.e., γ-glutamyl transferase (GGT) (16)], and antioxidant micronutrients (i.e., bilirubin, carotenoids, uric acid, vitamins A, C, D, and E) to the *sleep duration*–cardiometabolic health and *sleep quality*–cardiometabolic health relationships (14, 15). We did not, however, assess the contributions of CRP, GGT, and antioxidant micronutrients on sleep–insulin sensitivity or –glycemic control relationships. To date, only Kim et al. (17) has explored the association between sleep and markers of insulin resistance in an apparently healthy population; using a community-based sample of 374 participants, they found that the association between actigraphy-based sleep onset latency and HOMA-IR was partially mediated by CRP and the inflammatory cytokine, interleukin-6. What remains unclear, is the extent to which these factors may act collectively to augment, or offset, the detrimental effect of poor sleep hygiene on markers of metabolic disease or type 2 diabetes risk.

The purpose of our study is to quantify the contributions of CRP, GGT, and antioxidant micronutrients (i.e., bilirubin, carotenoids, uric acid, and vitamins A, C, D, and E) to the relationships between sleep and fasting insulin concentration or glycemic control, and thereby determine the extent to which they lie on the pathway. In this study, we use blood markers of inflammation, oxidative stress, and antioxidant micronutrients, rather than self-reported dietary data to minimize recall and healthy responder bias, and leverage data from a sample of nearly 2,000 free-living adults from the U.S.

## Methods

### Study Design, Setting, and Participants

Data for this analysis were obtained from the U.S. National Health and Nutrition Examination Survey (NHANES), a nationally representative cross-sectional study designed to assess the health and nutritional status of its non-institutionalized civilian population (18). Briefly, NHANES samples approximately 10,000 people bi-annually, collecting data from personal interviews, standardized physical examinations, and laboratory samples. While the most recent NHANES cycles (2019–2020) provide a comprehensive assessment of nutrition and many subjective and laboratory measures of health, NHANES 2005–2006 is the last cycle to include measures such as vitamins A, E, and carotenoids data that are relevant to the current analysis. Drawing from an initial sample of 10,348 NHANES 2005–2006 participants, exclusions for age (<20 y; *N* = 5,369), pregnancy (*N* = 336), and missing sleep/exposure (*N* = 2,571), mediators (*N* = 118), and outcomes (*N* = 8) were made in sequence, resulting in the final analytic sample of 1,946.

### Exposures: Sleep Duration and Quality

The Sleep Disorders Questionnaire was administered to NHANES participants aged ≥16 y, who reported their typical sleep habits on weekdays or workdays for the past month (18), and we used data from those aged ≥20 y in the present analysis. This Sleep Disorders Questionnaire contains items from two previously validated sleep questionnaires, but it has not been validated in its entirety (19, 20). A single question was used to collect sleep duration information: “*How much sleep do you usually get on weekdays or workdays?”* Responses to this question were collected in whole numbers between 1 and 11 h, and truncated at ≥12 h. Consistent with previous literature, sleep duration was categorized as “very short” (≤ 4 h), “short” (5–6 h), “adequate” (7–8 h), and “long” (≥9 h) (14).

Overall sleep quality was determined from the following 6 questions: “*How often did you have trouble falling asleep?;” “How often did you wake up during the night and had trouble getting back to sleep?;” “How often did you wake up too early in the morning and were unable to get back to sleep?;” “How often did you feel unrested during the day, no matter how many hours of sleep you had?;” “How often did you feel excessively or overly sleepy during the day?;”* and “*How often did you not get enough sleep?”* (18). Responses to these questions [0 = Never; 1 = Rarely (1 time a month); 2 = Sometimes (2–4 times a month); 3 = Often (5–15 times a month); and, 4 = Almost always (16–30 times a month)] were summed to obtain an overall sleep quality score (15, 18, 21). The sleep quality score was subsequently categorized as: “good” (0 to <3); “fair” (3 to <7); “poor” (7 to <12); and “very poor” (≥12 to 24) on the basis of previous work (15, 21).

### Outcomes: Fasting Insulin Concentration and Glycosylated Hemoglobin Levels

Fasting samples were obtained during the morning examination session after an overnight fast, and standard laboratory methods were used to quantify fasting insulin concentration (pM) and glycosylated hemoglobin (HbA1c, %), a marker for the last 2–3 months of glycemic control (18).

### Mediators: Inflammation, Oxidative Stress, and Antioxidant Micronutrients

Additional laboratory measures included in this study were: C-reactive protein [CRP (nM), a marker of inflammation]; γ-glutamyl transferase [GGT (U/L), a marker of oxidative stress]; and, bilirubin (μM), carotenoids (μM), uric acid (μM), and vitamins A (μM), C (μM), D (μM), and E (μM), markers of antioxidant micronutrients (18). These mediators were modeled continuously to assess their contributions to the sleep–insulin concentration and sleep–glycemic control relationships.

### Demographic and Behavioral Characteristics

Other variables used to describe the study population were age (20 to <40 y, 40 to <65 y, and ≥ 65 y), sex (M, F), ethnicity (White, Black, Latin, and Others groups), education, income, smoking history, and alcohol intake. Educational attainment was categorized as < high school, high school, college; and income as < $20,000, $20,000–44,999, and ≥$45,000. Smoking history was categorized as current (if smoking now), past (if smoked ≥100 cigarettes in one's life but not a current smoker), or never (if smoked <100 cigarettes in one's life) (14). Alcohol intake was categorized as none, <3, and ≥3 drinks per day.

### Mediation Model

**Figure 1:**
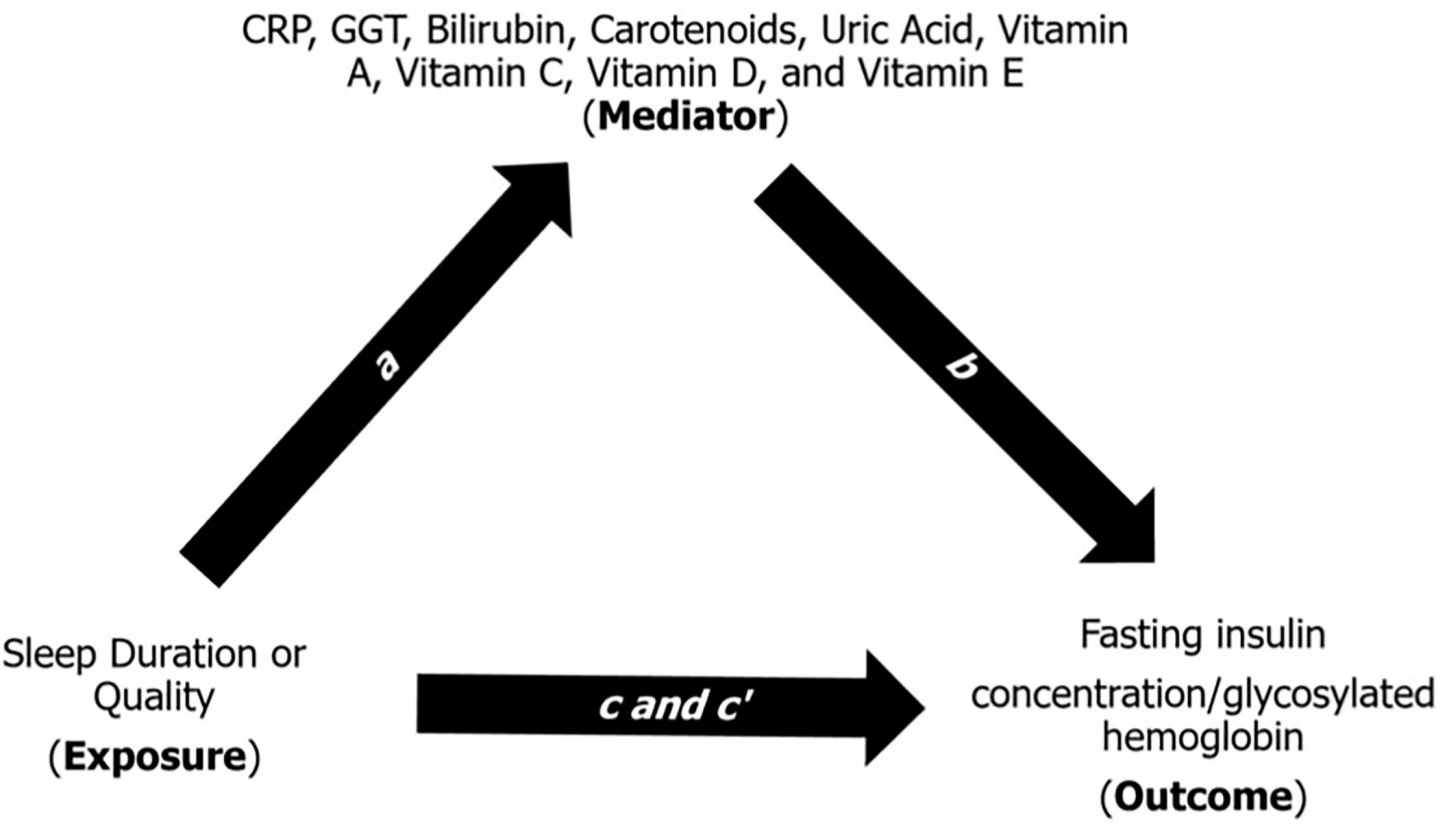
Multiple regression method of the indirect mediation model (22). Path *a* is a regression analysis between the exposure (e.g., sleep duration or quality) and the mediator (e.g., CRP, GGT, bilirubin, carotenoids, uric acid, and vitamins A, C, D, and E). Path *b* is a regression analysis between the mediator and the outcome (e.g., fasting insulin concentration) adjusting for the exposure. Path *c* is a regression analysis between the exposure and outcome. Path *c'* is a regression analysis between the exposure and the outcome adjusting for the mediator. The indirect effect (*ab*) estimate is the amount of contribution a mediator provides to the relationship between an exposure and an outcome. CRP, C-reactive protein; GGT, gamma-glutamyl transferase. Each mediator was tested one-by-one in the mediation model.

The mediation model helps explain the underlying relationship between an exposure variable and an outcome measure through a third (mediatory) variable (22). Briefly, the mediation model (Figure 1) is a series of regression analyses that contains four path analyses: (1) path *a* is a regression between the exposure and the mediator; (2) path *b* is a regression between the mediator and the outcome while adjusting for the exposure; (3) path *c* is a regression between the exposure and the outcome; and (4) path *c'* is a regression between the exposure and the outcome while adjusting for the mediator. In the mediation model, the products of *ab* and *c-c'* are mathematically equivalent, and *ab* is considered as the “amount” of mediation or contribution a mediator provides to the relationship between an exposure and an outcome. As described by Kenny (22), the contribution of each mediator can also be described as “large” (*ab* ≥ 0.25), “moderate” (*ab* ≥ 0.09), “modest” (*ab* ≥ 0.01), and “weak” or “modest” (*ab* < 0.01). We further used multiple comparisons correction, i.e., Bonferroni (*p* = 0.05/9) and a *p*-value generated through simulation, to interpret our indirect effect given that it is a common practice in mediation analysis in the fields of genetics and metabolomics (23, 24).

### Statistical Analyses

Mean and 95% confidence interval (CI) for continuous variables, and frequency (percentage), and 95% CI for categorical variables were stratified by sleep duration. ANOVA and χ^2^ tests were used, as appropriate, to test for any differences in demographic and behavioral characteristics across groups. The medical exam sample weight from the demographics data file was used to weight descriptive analyses to be representative of the US adult population (18). For the mediation analysis, we used Hayes' INDIRECT SAS Macro with the bootstrap method with 5,000 iterations to estimate the amount of mediation or contribution (*ab*) by each mediator, and present the bias corrected *ab* estimates with 95% CI, and *p*-values (25, 26). All analyses were conducted in SAS v9.3 (Cary, NC, USA), and statistical significance was set at an α of 0.05.

## Results

### Demographic and Behavioral Characteristics

Table 1 describes the sample characteristics by sleep duration. Very short sleep was higher amongst 40–65-years-old, men, Black ethnicity, lower income, and current smokers. On the other hand, a higher proportion of adequate and long sleepers were found among White ethnicity and in those with a college education. As expected, a greater proportion of participants reporting shorter sleep duration also reported lower sleep quality, but approximately a third of the participants reporting adequate and long sleep still reported poor sleep quality.

**Table 1:** Characteristics of the US adult population ≥20 years of age by sleep duration.

**Characteristics**		**Sleep duration per night**				***p*-value**
		**Very short (*n* = 113)**	**Short (*n* = 662)**	**Adequate (*n* = 1,013)**	**Long (*n* = 158)**	
Age [Mean (95% CI)]		45.2 (40.4, 50.0)	46.1 (44.4, 47.9)	47.8 (46.0, 49.7)	48.4 (43.8, 53.0)	<0.05
Age categories [% (95% CI)]					
	20 to <40 years	32.7 (15.6, 49.8)	36.4 (31.9, 40.9)	35.7 (32.3, 39.2)	38.1 (30, 46.3)	0.002
	40 to <65 years	57.9 (43.3, 72.5)	49.1 (45.3, 53)	45.6 (42.4, 48.8)	34.4 (23.4, 45.5)	
	≥65 years	9.4 (4.4, 14.4)	14.4 (10.1, 18.8)	18.7 (14.5, 22.9)	27.4 (16.9, 38.0)	
Gender						
	Men	56.9 (41.6, 72.2)	53.6 (50.4, 56.8)	49.9 (46.7, 53.1)	37.2 (30.1, 44.2)	0.011
	Women	43.1 (27.8, 58.4)	46.4 (43.2, 49.6)	50.1 (46.9, 53.3)	62.8 (55.8, 69.9)	
Ethnicity						
	White	64.3 (52.1, 76.6)	65.5 (57.5, 73.5)	76.1 (70.2, 81.9)	75.7 (67.0, 84.5)	<0.001
	Black	22.2 (11.6, 32.8)	16.8 (11.3, 22.3)	7.6 (4.4, 10.7)	8.4 (5.5, 11.3)	
	Latin	6.4 (3.3, 9.5)	7.6 (5.5, 9.8)	7.5 (5.0, 10.1)	7.0 (3.6, 10.4)	
	Others	7.1 (0, 15.5)	10.0 (6.1, 13.9)	8.8 (4.7, 12.9)	8.8 (2.0, 15.7)	
Education						
	< High school	23.1 (13.5, 32.6)	15.3 (11.2, 19.5)	17 (12.7, 21.3)	20.6 (12.7, 28.6)	0.063
	High school	35.4 (20.2, 50.7)	28.0 (24.6, 31.5)	23.6 (19.9, 27.4)	22.4 (17.8, 27.1)	
	College	41.5 (26.0, 57.0)	56.6 (50.9, 62.3)	59.4 (53.0, 65.8)	56.9 (45.6, 68.3)	
Income						
	< $20,000	26.2 (17.0, 35.4)	16.7 (12.6, 20.7)	13.3 (10.0, 16.6)	19.6 (13.3, 25.8)	0.001
	$20,000–44,999	34.0 (24.4, 43.7)	31.3 (26.0, 36.7)	30.4 (25.2, 35.6)	36.2 (27.7, 44.7)	
	≥$45,000	39.8 (30.8, 48.8)	52.0 (44.0, 60.0)	56.3 (49.9, 62.7)	44.2 (36.8, 51.6)	
Smoking						
	None	36 (27.1, 44.8)	47.3 (39.7, 54.8)	49.2 (43.7, 54.7)	53 (43.9, 62.1)	<0.001
	Current	51.3 (41.6, 61.0)	30.5 (22.5, 38.5)	21.3 (16.8, 25.8)	25.5 (18.0, 33.0)	
	Past	12.7 (4.7, 20.8)	22.2 (17.1, 27.3)	29.5 (25.9, 33.1)	21.5 (13.9, 29.1)	
Alcohol intake						
	None	6.6 (4.9, 8.3)	33.9 (29.4, 38.4)	52.0 (47.7, 56.2)	7.5 (5.7, 9.3)	0.663
	<3 drinks per day	4.6 (2.5, 6.7)	29.8 (24.6, 34.9)	57.6 (52.1, 63.0)	8.1 (5.1, 11.1)	
	≥3 drinks per day	5.5 (2.4, 8.6)	33.0 (24.0, 42.0)	55.1 (48.4, 61.8)	6.3 (3.6, 9.1)	
Sleep quality						
	Good	1.4 (0, 3.2)	9.6 (6.2, 13.1)	18.6 (14.5, 22.8)	21.0 (16, 26.1)	<0.001
	Fair	11.0 (2.0, 20.0)	16.3 (13.2, 19.4)	29.2 (25.7, 32.6)	31.1 (22.1, 40.0)	
	Poor	14.2 (5.1, 23.4)	30.6 (25.0, 36.2)	35.2 (31.7, 38.6)	33.5 (24.5, 42.5)	
	Very poor	73.4 (63.9, 82.9)	43.5 (36.0, 50.9)	17.0 (13.3, 20.7)	14.4 (7.7, 21.1)	

*Mean (95% CI) for continuous variables and % (95% CI) for categorical variables. Sleep Duration Categories: Very Short (≤ 4h per night), Short (5-6h per night), Adequate (7-8h per night), and Long (≥9h per night). Responses to six sleep habits questions were summed and categorized into quartiles: Good (<3), Fair (≥3 to 7), Poor (≥7 to 12), and Very Poor (≥12). p <0.05, two-sided; ANOVA or Chi-square, as appropriate. Sum of weights = 91,008,644*.

### Estimates of Mediations or Contributions

**Figure 2:**
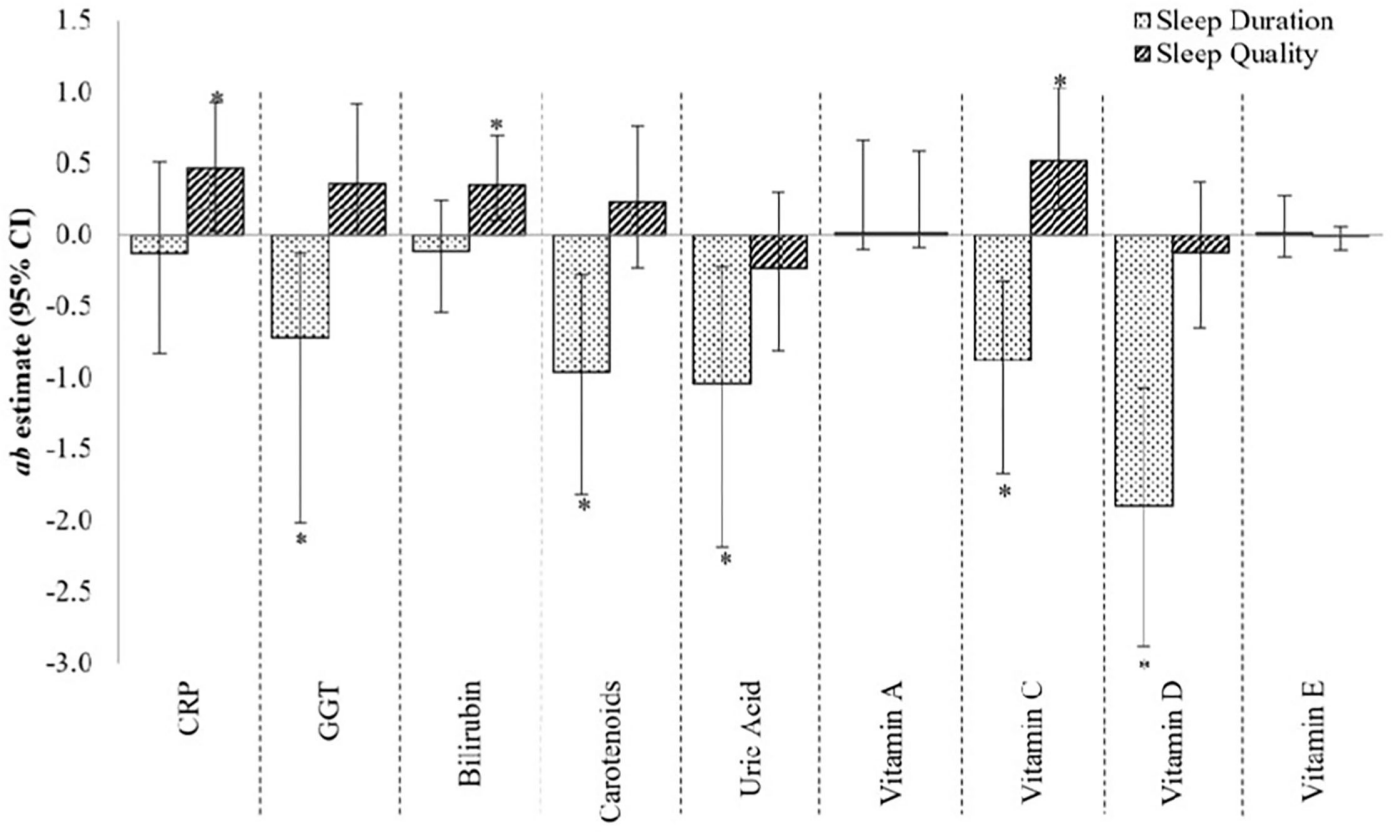
The contribution of CRP, GGT and micronutrient antioxidants to the sleep–fasting insulin concentration relationship. *ab* estimate, amount of mediation, or contribution by the mediatory variable (moderate effect when *ab* ≥ 0.09, and large effect when *ab* ≥ 0.25); CI, confidence interval. *p <0.05, 95% CI are bias-corrected, bootstrapped values. Each mediator was tested one-by-one in the mediation model.

Figure 2 provides the estimates of mediation or contribution by each mediatory variable (i.e., CRP, GGT, bilirubin, carotenoids, uric acid, and vitamins A, C, D, and E) to the sleep–fasting insulin concentration relationship. GGT, carotenoids, uric acid, and vitamins C and D provided large and statistically significant contributions to the *sleep duration*–fasting insulin concentration relationship [*ab* estimate (95% CI), *p*-value: −0.73 (−2.02, −0.13), 0.03; −0.97 (−1.82, −0.28), 0.02; −1.04 (−2.19, −0.22), 0.02; −0.88 (−1.67, −0.33), 0.01; and −1.90 (−2.89, −1.07), <0.01, respectively]. Additionally, though non-significant, CRP and bilirubin were moderate contributors to the sleep duration–fasting insulin concentration [−0.13 (−0.83, 0.51), 0.70; and −0.12 (−0.55, 0.24), 0.54, respectively], while vitamins A and E contributed only modestly [0.01 (−0.11, 0.66), 0.79; and 0.01(−0.16, 0.27), 0.90, respectively] to the same relationship. The total effect of sleep duration–fasting insulin concentration relationship was −2.56 (SE: 2.56; *p*-value: 0.32).

For the relationship between *sleep quality* and fasting insulin, CRP, bilirubin, and vitamin C made large and statistically significant contributions [0.47 (0.02, 0.92), 0.05; 0.34 (0.09, 0.69), 0.03; and 0.51 (0.17, 1.03), 0.01, respectively] (Figure 2), whereas GGT made a large but non-significant contribution [0.35 (0.00, 0.92), 0.09]. Carotenoids, uric acid, and vitamin D, on the other hand, made moderate, non-significant contributions [0.23 (−0.23, 0.76), 0.36; −0.23 (−0.81, 0.29), 0.42; and −0.13 (−0.65, 0.36), 0.63, respectively] to the sleep quality–fasting insulin concentration relationship, while vitamin A contributed only modestly [0.01 (−0.09, 0.58), 0.82]. The total effect of sleep quality–fasting insulin concentration relationship was 2.46 (SE: 1.68; *p*-value: 0.14).

**Figure 3:**
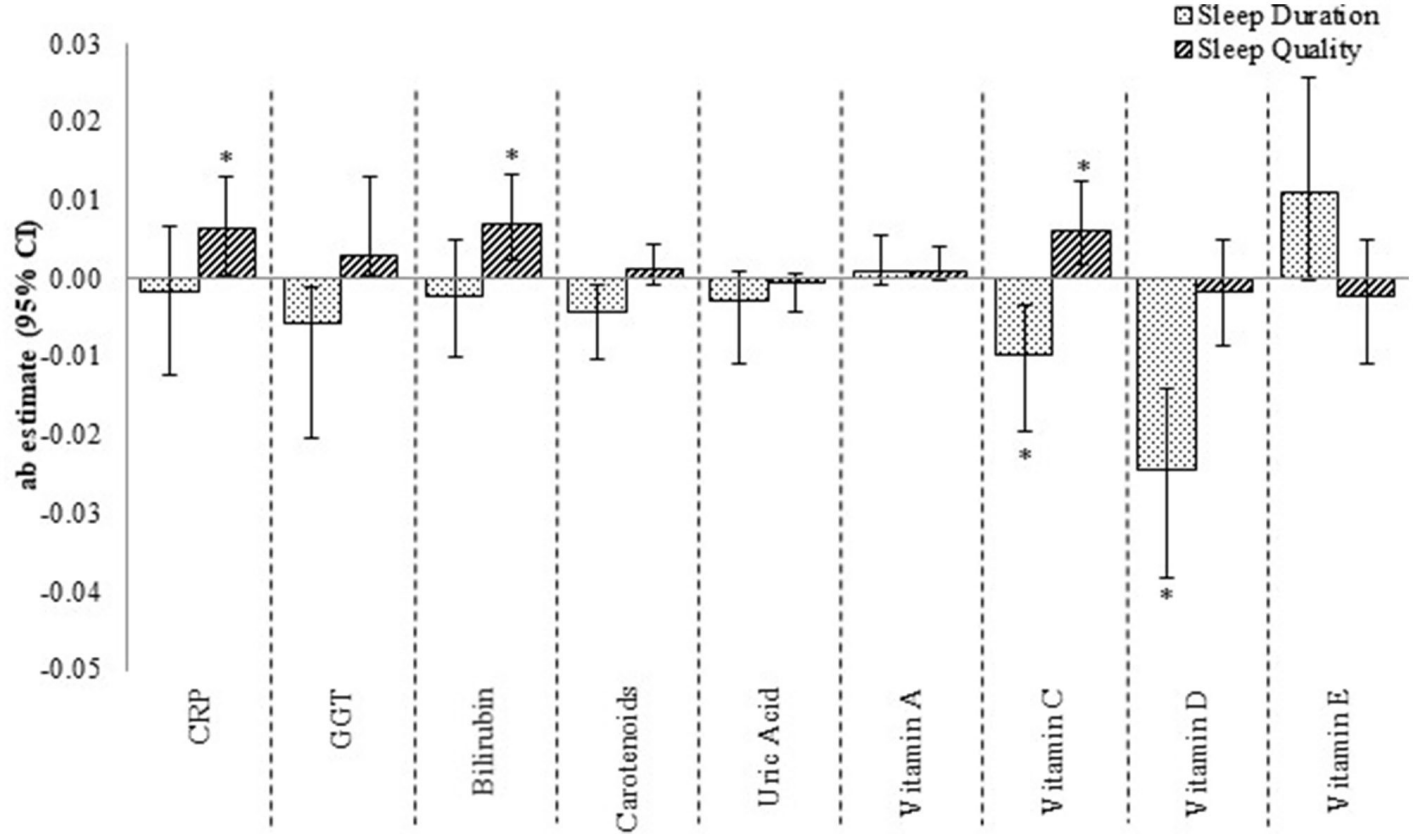
The contribution of CRP, GGT and micronutrient antioxidants to the sleep–glycosylated hemoglobin relationship. *ab* estimate, amount of mediation or contribution by the mediatory variable (moderate effect when *ab* ≥ 0.09, and large effect when *ab* ≥ 0.25) CI, confidence interval. *p <0.05, 95% CI are bias-corrected, bootstrapped values. Each mediator was tested one-by-one in the mediation model.

The mediating effect of these micronutrients was only weak-to-modest when the outcome was HbA1c (Figure 3). Vitamins C and D, for instance, were significant but modest contributions to the *sleep duration*–glycosylated hemoglobin relationship, while CRP, bilirubin, and vitamin C were modest, non-significant mediators of the *sleep quality*–glycosylated hemoglobin relationship. The total effect of sleep duration–glycosylated hemoglobin and sleep quality–glycosylated hemoglobin relationships were −0.07 (SE: 0.03; *p*-value: 0.04) and 0.02 (SE: 0.02; *p*-value: 0.41), respectively.

When we used the Bonferroni corrected *p*-value of <0.0056, the only significant mediator of sleep duration–fasting insulin concentration and –glycosylated hemoglobin relationships was Vitamin D. None of our mediators contributed significantly to the relationships between sleep and insulin sensitivity or glycosylated hemoglobin if we used the simulated *p*-value of <0.0001 for multiple biological mediators testing.

## Discussion

Our aim was to quantify the contribution of (CRP, GGT, bilirubin, carotenoids, uric acid, and vitamins A, C, D, and E) to the sleep–fasting insulin concentration and –glycemic control relationships, and thus, determine whether inflammation, oxidative stress, and antioxidant micronutrients lie on the pathway and act as mediators of these relationships. In this regard, we found that GGT, carotenoids, uric acid, and vitamins C and D contributed significantly to the relationship between *sleep duration* and fasting insulin concentration, while CRP, bilirubin, and vitamin C contributed significantly to the relationship between *sleep quality* and fasting insulin concentration. While significant, these factors were more modest mediators of the sleep–glycemic control relationship. Our findings suggest that poor sleep quality and/or duration may impact various biochemical analytes associated with oxidative stress and inflammation that may in turn promote whole body insulin resistance, particularly in middle-aged females. To our knowledge, this is the first time that the mediation effect of inflammation, oxidative stress, and antioxidant micronutrients on measures of glycemic control have been evaluated in a representative U.S. sample, and several of our findings warrant discussion.

To date, relatively few studies have examined the inter-relationship between inflammation, oxidative stress, and antioxidant micronutrients on the pathway between sleep and diabetes risk (8, 9, 14, 15, 17). Of these, our study is most comparable to Kim et al.'s (17) community-based cross-sectional study, which found partial mediation by CRP and interleukin-6 on the relationship between sleep onset latency and HOMA-IR. Main differences between this study and ours, includes Kim's use of actigraphy-based sleep measures, adjustment for diabetes and depression, and investigation of moderation by sex. Our analysis accounts for a diverse range of age, demographic, and ethnic groups while investigating diabetes-related outcomes, building upon previous analyses by our group that included sex-stratified results (14).

While it is not clear why the contributions may be stronger in women, sleep disturbance induced psychological stress and physiological changes may provide a partial explanation (27). Indeed, age, sex, smoking history, alcohol consumption, physical activity, and comorbidities are known confounders of the multiple linear relationships evaluated in the present mediation analysis (8, 28–32). For instance, moderate intensity and lifestyle-related physical activity have been found to be large mediators of the sleep–fasting insulin concentration relationship (28). It is well known that physical activity plays an important roles in glycemic control due to its acute blunting effect on insulin levels (33), and that muscular contraction stimulates insulin sensitivity by increasing AMPK activity, deactivating TCB1D1 and promoting GLUT4 translocation to the cell membrane, which increases cellular glucose uptake (34). Therefore, future attempts to quantify, and de-couple the direct and indirect paths between sleep and metabolic health would benefit from causal modeling that considers a more comprehensive set of factors (as covariates. confounders, mediators, and moderators).

Finally, in exploring mediators of the relationship between sleep, insulin sensitivity, and glycemic control, we are attempting to understand factors which underlie the pathway as possible mechanistic links. It therefore bears repeating that although we used Preacher and Hayes's (25). SAS procedure to estimate the indirect effects (path *ab*), the negative *ab* does not indicate a negative mediation, but rather that one of the paths is negative while the other is positive (22). For example, since the association between sleep duration and CRP (path *a*) is negative, and the relationship between CRP and fasting insulin concentration (path *b*) is positive (35), the resulting *ab* is negative. Similarly, since the association between sleep quality and CRP is positive (i.e., path *a* and path *b* is also positive), the resulting *ab* product is positive. Our finding of a much weaker mediation by CRP, bilirubin, and vitamins C and D to the sleep–glycosylated hemoglobin relationship (as compared to insulin sensitivity) also warrants discussion, and may be explained by the use of the bootstrap method—a non-parametric test that assumes linear relationships between paths—which likely produced conservative estimates of the indirect effect sizes (25), or a narrower homeostatic range of glycosylated hemoglobin in the non-diabetic and medicated diabetic populations (36).

## Limitations

There are several limitations associated with our study. First, though mediation analysis is a causal path analysis, given the cross-sectional nature of the design, causal inference may not be made from our findings alone, and future longitudinal studies are needed to confirm the directionality and importance of these findings. Second, in applying our study exclusion criteria, our final analytic sample was only a fraction of the initial adult sample; however, all analyses were bootstrapped with replacement, which provided conservative, bias-corrected indirect effect estimates. Given that our study considered multiple mediators, the use of a lower *p*-value for significance testing and estimating a narrower confidence interval—a common practice in the genetics literature (23)—may be a reasonable alternative for evaluating the indirect effects. Further, mediators and outcomes were objectively measured, but sleep measures were self-reported and susceptible to recall and response bias.

## Conclusions

CRP, GGT, bilirubin, carotenoids, uric acid, and vitamins C and D made large, significant contributions to the observed relationships between sleep and fasting insulin concentration, but explained less of the relationship with glycosylated hemoglobin. Since the mediators explored in this study are linked to diet, physical activity, and sleep behaviors, interventions that collectively target modifiable lifestyle behaviors including sleep hygiene, reduce systemic inflammation/oxidative stress, and optimize antioxidant intake, remain mutually beneficial targets for diabetes risk.

## Data Availability Statement

Publicly available datasets were analyzed in this study. This data can be found at: https://www.cdc.gov/nchs/nhanes/index.html.

## Author Contributions

TK designed the study, conducted the analyses, and drafted the manuscript. MR and CA consulted on the study design, analysis, interpretation of findings, and revised the drafted manuscript. All authors reviewed and approve of the final manuscript version.

## Conflict of Interest

The authors declare that the research was conducted in the absence of any commercial or financial relationships that could be construed as a potential conflict of interest.

## Publisher's Note

All claims expressed in this article are solely those of the authors and do not necessarily represent those of their affiliated organizations, or those of the publisher, the editors and the reviewers. Any product that may be evaluated in this article, or claim that may be made by its manufacturer, is not guaranteed or endorsed by the publisher.
